# Metabolism and Targeted Therapy of Fibrosis in Chronic Pancreatitis: A Review

**DOI:** 10.7150/ijms.118338

**Published:** 2025-07-28

**Authors:** Hongqing Luo, Shan Guo, Yuning Chu, Yiping Xin, Xiaoyan Yin, Xiaoyu Li

**Affiliations:** Department of Gastroenterology, The Affiliated Hospital of Qingdao University, Qingdao, China.

**Keywords:** chronic pancreatitis, pancreatic fibrosis, metabolic reprogramming, pancreatic stellate cells, glycolysis, therapeutic strategies

## Abstract

Chronic pancreatitis (CP) is a progressive condition characterized by persistent pancreatic inflammation, tissue destruction, and fibrosis. Recent studies have highlighted the crucial role of metabolic processes in the pathogenesis of pancreatic fibrosis, particularly the metabolic reprogramming of pancreatic stellate cells (PSCs) and immune cells. Disruptions in glucose, lipid, and amino acid metabolism have been shown to play a key role in the progression of CP fibrosis, exacerbating disease severity. Activated PSCs exhibit enhanced glycolysis and lipid metabolism, which promote excessive extracellular matrix (ECM) production and tissue remodeling. Simultaneously, immune cells such as macrophages and T cells undergo metabolic reprogramming, further intensifying inflammation and fibrosis. This review discusses the role of metabolic reprogramming in pancreatic fibrosis and proposes potential therapeutic strategies targeting metabolic pathways, including glycolysis inhibitors, lipid metabolism modulators, and amino acid metabolism regulators. These strategies offer promising prospects for mitigating the progression of CP fibrosis and provide new therapeutic avenues for clinical applications.

## 1. Introduction

Chronic pancreatitis (CP) is characterized by prolonged inflammation in the pancreas, resulting in tissue damage and fibrosis. The disease has diverse etiologies, including alcohol abuse, biliary obstruction, and—in a small subset of cases—hereditary mutations such as PRSS1, SPINK1, or CFTR 1. Regardless of etiology, sustained pancreatic injury leads to chronic inflammation and progressive fibrosis, which are closely linked to immune-metabolic dysregulation.

Early in the disease, repeated inflammatory episodes in pancreatic tissue gradually lead to fibrosis, severely impairing pancreatic structure and function 2. This fibrosis eventually results in the decline of both endocrine and exocrine functions, affecting digestion and glucose regulation in patients and potentially leading to more severe complications such as pancreatic cancer 3.

Mechanistically, injured acinar cells release signals that prompt the recruitment of immune cells, triggering inflammatory responses and differentiation processes 4. Due to their significant plasticity and heterogeneity, macrophages originate from bone marrow-derived monocytes, and their diverse phenotypes play crucial roles in both the onset and persistence of tissue damage and fibrosis 5. Activated T cells have different cell subsets and can secrete various cytokines, which play a key role in the inflammation and fibrosis of CP 6. Pancreatic stellate cells (PSCs) are a type of mesenchymal cell located within the pancreatic tissue, where they help preserve normal physiological structure and function. Signals stemming from injury and immune cells activate PSCs, transforming them into a myofibroblast-like phenotype, which is a critical step in the progression of pancreatic fibrosis 7. Upon activation, PSCs proliferate extensively and produce excessive extracellular matrix (ECM) components. Consequently, macrophages, T cells, and PSCs adapt their metabolism to meet the increased energy demands and the need for raw materials to sustain these physiological activities. The excessive ECM accumulation and cross-linking that ensue eventually compromise the cellular microenvironment, leading to the disruption of structural integrity and homeostasis within the pancreas. Ultimately, pancreatic fibrosis is associated with high morbidity and mortality due to pancreatic failure and its potential progression to pancreatic cancer 8.

Pancreatic fibrosis stands out as a hallmark of chronic pancreatitis and is a significant contributor to the long-term complications of the disease 9, 1. Various therapeutic strategies have been explored in recent years, including antioxidant therapy, gene therapy, and immunotherapy 6, 10. For a small subset of CP cases that is genetically determined, targeting the underlying disease-predisposing or -causing mutations may be a crucial therapeutic strategy 10. However, most of these therapeutic approaches have had limited efficacy in halting or reversing fibrosis. Therefore, greater attention has been directed toward antifibrotic therapies, particularly those that target the underlying cellular and molecular mechanisms of fibrosis.

This article emphasizes the connection between metabolic regulation and pancreatic fibrosis. The metabolic states of immune cells and PSCs are closely intertwined with their functional capacities 11. Factors such as nutrient availability, redox balance, gut-derived metabolites and microbiota, and circadian rhythms modulate cellular metabolism, ultimately determining cellular function. Similarly, alterations in cellular functions driven by immune or growth factor signals necessitate adjustments in metabolism to fulfill these demands 12. Therefore, targeting immune cell and PSC metabolism represents a promising therapeutic strategy in addressing pancreatic fibrosis.

While existing reviews have discussed immune cell function or PSC activation in CP, few have specifically focused on the metabolic reprogramming shared across immune and stromal cells and its role in driving fibrogenesis 13, 6. This review aims to fill this gap by providing an integrative perspective on how metabolism links inflammation and fibrosis in CP and how these insights may inform more precise anti-fibrotic interventions. Recognizing the influence of metabolic reprogramming on pancreatic fibrosis may pave the way for new treatments for chronic pancreatitis.

## 2. Acinar Cell Damage and Oxidative Stress

Metabolic disturbances play a fundamental role in the pathogenesis of fibrosis during chronic pancreatitis. Normally, pancreatic cells utilize various metabolic pathways, including glucose, lipid, and amino acid metabolism, to support their functions. However, in the presence of chronic inflammation and changes in the immune microenvironment, both recruited and resident immune cells, alter their metabolic activity and prioritize specific pathways to adapt to the demands of survival, proliferation, and function 14, 15. Acinar cell damage is recognized as a key trigger in chronic pancreatitis. In patients with CP, the expression of interferon-γ-inducible protein 10 (CXCL10) in pancreatic tissue is upregulated. CXCL10 can induce apoptosis and DNA damage through C-X-C motif chemokine receptor 3 (CXCR3) signaling. Upon stimulation by CXCL10, the expression of cytochrome C, Apaf-1, and caspase 3/9 is upregulated in acinar cells, leading to apoptosis. Concurrently, there is a change in membrane potential, mitochondrial dysfunction occurs, and ATP is significantly depleted 16. Mitochondrial dysfunction leads to ATP depletion, which in turn causes an accumulation of Ca^2+^ in the cytoplasm. Pathological Ca^2+^ signaling activates the calcium-dependent phosphatase calcineurin, resulting in the premature activation of trypsinogen 17. Trypsinogen, through a non-ATPase proteasome pathway, mediates the degradation of glutathione peroxidase 4, thereby increasing the sensitivity of acinar cells to ferroptosis and triggering pancreatitis 18. Estrogen-related receptor γ (ERRγ) is vital for maintaining mitochondrial oxidative phosphorylation (OXPHOS) in pancreatic acinar cells, and its downregulation can lead to mitochondrial dysfunction, energy depletion, increased reactive oxygen species (ROS) accumulation, and progressive pancreatic atrophy, thereby exacerbating pancreatitis. In patients with CP, ERRγ expression is downregulated, and polymorphisms in the ESRRG gene, which encodes ERRγ, as well as single nucleotide variants, have been linked to CP 19.

ROS are byproducts of oxidative metabolism. An imbalance between ROS production and degradation can lead to oxidative stress, which may trigger chronic pancreatitis 20. ROS regulate the expression of nuclear factor kappa B (NF-κB), which in turn induces the expression of transforming growth factor β (TGF-β) and fibrosis-related genes 21. TGF-β activates PSCs, leading to the production of extracellular matrix proteins and the promotion of pancreatic fibrosis 22. Excessive consumption of carbohydrates and fats enhances mitochondrial oxidative respiration, significantly elevating ROS levels. This increase in ROS triggers lipid peroxidation, resulting in the substantial accumulation of metabolites like malondialdehyde (MDA) within the body, which in turn disrupts lipid metabolism in the pancreas 23, 24. Elevated glucose concentrations enhance the production of ROS in PSCs, thereby driving their activation and contributing to the progression of fibrosis 25. Therefore, it is essential to acknowledge the pivotal role of metabolic pathways in driving the progression of chronic pancreatitis.

## 3. The Role of Immune Cell Metabolic Reprogramming in Fibrosis

### 3.1 Macrophage Metabolic Reprogramming

Macrophages, monocytes derived from bone marrow monocytes or yolk sac cells during embryonic development, are responsible for the removal of dead or harmful pathogens and play an important role in inflammation, tissue repair, and homeostasis 26 27. Macrophages can be divided into M1 macrophages (classical activated type) and M2 macrophages (alternative activated type) according to their different phenotypic functions 28. Under normal physiological conditions, resident macrophages are sparse in the pancreas, proliferating, polarizing, and repairing tissue only when pathological changes occur. Initially, they respond to the local environment in a pro-inflammatory manner, but they rapidly transition to a wound repair phenotype. The immune characteristics of macrophages differ across CP subtypes. For example, idiopathic CP presents a higher prevalence of CD68^+^ macrophages compared to hereditary CP. Idiopathic CP also shows increased expression of interleukin-4 (IL-4) and IL-13, whereas hereditary CP shows elevated tumor necrosis factor-α (TNF-α) and IL-6 levels 29.

**Figure 1:**
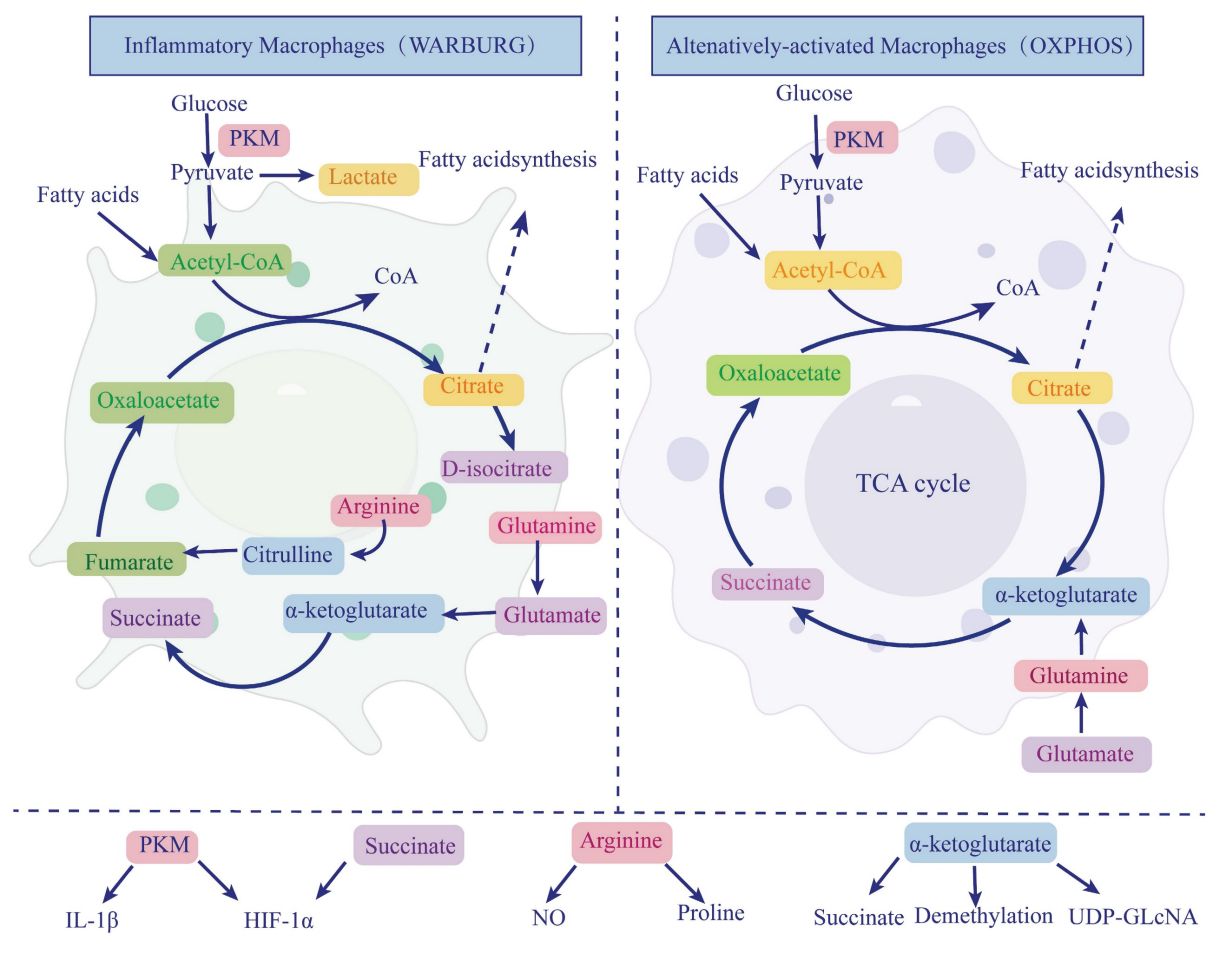
** Macrophage metabolic reprogramming in chronic pancreatitis.** Macrophages exhibit distinct metabolic phenotypes in chronic pancreatitis (CP). Classically activated macrophages (M1) primarily rely on glycolysis, even in the presence of oxygen, a phenomenon similar to the Warburg effect. This shift leads to the production of lactate and the interruption of the tricarboxylic acid (TCA) cycle at two key nodes, resulting in the accumulation of citrate and succinate. Succinate stabilizes hypoxia-inducible factor 1-alpha (HIF-1α), which in turn promotes the transcription of proinflammatory cytokines such as IL-1β. Additionally, pyruvate kinase M2 (PKM2) enhances HIF-1α and IL-1β expression, reinforcing the inflammatory phenotype of M1 macrophages. In contrast, alternatively activated macrophages (M2) utilize oxidative phosphorylation (OXPHOS) and fatty acid oxidation to support anti-inflammatory functions and tissue repair. In M2 macrophages, glutamine-derived α-ketoglutarate (α-KG) fuels the TCA cycle and serves as a substrate for epigenetic regulation via histone demethylation and UDP-GlcNAc synthesis, facilitating M2 polarization. Arginine metabolism also differs between subtypes: M1 macrophages convert arginine to nitric oxide (NO) to maintain their inflammatory role, whereas M2 macrophages convert arginine to proline, contributing to collagen synthesis and fibrosis progression. HIF-1α, hypoxia-inducible factor 1-alpha; OXPHOS, oxidative phosphorylation; PKM, pyruvate kinase M; α-KG, α-ketoglutarate; TCA, tricarboxylic acid cycle; NO, nitric oxide; UDP-GlcNAc, uridine diphosphate N-acetylglucosamine.

M1 macrophages predominantly infiltrate during the early stages of chronic pancreatitis. M0 macrophages migrating from the bone marrow polarize into M1 macrophages, releasing TGF-α/β, IL-6, and matrix metalloproteinase-10 (MMP-10), contributing to pancreatic inflammation and injury 30. In CP, M2 macrophages become the predominant infiltrating cells, mediating pancreatic fibrosis. Both infiltrating macrophages and PSCs secrete IL-6, which induces PSCs to produce TGF-β1 through IL-6R/STAT3 signaling, promoting PSC activation and fibrosis 31. Dachaihu decoction, a traditional Chinese medicine formula, has been shown to inhibit pancreatic macrophage infiltration, reduce IL-6 and chemokine expression (such as monocyte chemoattractant protein-1 (MCP-1) and macrophage inflammatory protein-1α (MIP-1α)), and improve pancreatic fibrosis 32. Moreover, M2 macrophages interact with PSCs, promoting pancreatic fibrosis by secreting TGF-β and platelet-derived growth factor β (PDGFβ), which further activate PSCs 26. When bone marrow-derived macrophages are co-cultured with PSCs, they exhibit increased expression of CD206, IL-10, TGF-β, and PDGFβ, while nitric oxide (NO) synthase expression decreases, indicating that PSCs can promote macrophage polarization to the M2 phenotype 33. PSCs secrete chemokine MCP-1, inducing alternative macrophage activation via NF-Κb 34. Pirfenidone reduces M2 macrophage infiltration in the pancreas and inhibits PSC cytokine release, decreasing both M2 macrophage and PSC activation, thereby alleviating pancreatic fibrosis 35. The physiological function of macrophages is closely related to fibrosis in chronic pancreatitis. Therefore, understanding macrophage metabolism can better explore the relationship between macrophages and CP [Figure 1].

#### 3.1.1 Glucose Metabolism

Macrophage glucose metabolism is central to their activation and function, particularly in different immune responses, where glucose metabolic pathways regulate macrophage phenotypes 36. In M1 macrophages, glycolysis serves as the primary energy source. Even under aerobic conditions, macrophages tend to favor anaerobic glycolysis, a pathway that provides quick energy while also enhancing the expression of MMP-1, IL-1β, and IL-6 in U937 macrophage-like cells via NF-κB and mitogen-activated protein kinase (MAPK) signaling 37, 38. This metabolic shift exacerbates inflammation in CP, as increased glycolysis is closely linked to maintaining and intensifying pro-inflammatory responses. The Warburg effect observed in M1 macrophages is similar to that in tumor cells, demonstrating a high dependence on hexokinase and glucose-6-phosphate dehydrogenase, further supporting their heightened metabolic activity and sustained inflammatory responses 39. In contrast, M2 macrophages rely more on OXPHOS for energy production 40. OXPHOS occurs through the mitochondrial electron transport chain, generating ATP more efficiently, giving M2 macrophages an advantage in tissue repair and anti-inflammatory responses 41.

In M1 macrophages, HIF-1α is upregulated, while in M2 macrophages, HIF-2α activation induces the expression of arginase 1 (ARG1), which suppresses NO production 42. Neddylation, a key post-translational modification, can be targeted with small-molecule inhibitors such as MLN4924, which inactivates the neddylation pathway, promoting HIF-1α-mediated chemokine ligand 5 (CCL5) secretion and enhancing M2 macrophage infiltration in chronic pancreatitis 43. Pyruvate kinase M2 (PKM2) is a key regulator of the Warburg effect in LPS-activated macrophages, modulating HIF-1α activity and IL-1β induction 44. PKM2 interacts with Smad7, interfering with Smad7 and TGFβR1 interaction and affecting the activation of the TGFβ1 signaling pathway. The tetrameric form of PKM2 helps regulate the TGFβ1 signaling pathway balance, promoting fibrosis 45. PKM2 also acts as a co-activator of HIF-1α, stimulated by prolyl hydroxylases 3 (PHD3) 46. Additionally, succinate accumulation inhibits PHDs, preventing them from hydroxylating HIF-1α, leading to HIF-1α stabilization 47. PKM2 further regulates glycolysis in LPS-activated macrophages by controlling lactate-induced K62 lactylation, facilitating the transition from a pro-inflammatory phenotype to a reparative phenotype, and accelerating wound healing 48.

In summary, macrophage glucose metabolism regulates energy production and biosynthesis, finely tuning their functions and phenotypes in different immune environments. Modulating macrophage glucose metabolism could potentially control the excessive activation of M1 macrophages, alleviating pancreatic inflammation and slowing disease progression.

#### 3.1.2 Amino Acid Metabolism

Amino acid metabolism is also crucial for macrophage function. Glutamine is one of the most important amino acids in macrophage metabolism, serving not only as a substrate for protein synthesis but also influencing macrophage activation and function through various pathways 49. In M1 macrophages, glutamine is converted into α-ketoglutarate, entering the Krebs cycle to support succinate production. Glutamine metabolism generates succinate, which can inhibit HIF-1α degradation, stabilizing its expression and promoting the pro-inflammatory functions of M1 macrophages.

Conversely, M2 macrophages utilize glutamine for oxidative phosphorylation and epigenetic reprogramming. The α-ketoglutarate (α-KG) produced from glutamine catabolism supports the anti-inflammatory phenotype of M2 macrophages and regulates gene expression through demethylation, promoting tissue repair functions 50, 40. Additionally, glutamine serves as a precursor for UDP-GlcNAc synthesis, participating in protein glycosylation in M2 macrophages, further enhancing their reparative functions 51. In chronic pancreatitis, imbalances in glutamine metabolism may lead to dysregulation of M1 and M2 macrophage functions, exacerbating inflammation or promoting fibrosis.

Other amino acids, such as arginine, also play critical roles in regulating macrophage phenotypes 52. M1 macrophages metabolize arginine to produce NO, enhancing bactericidal and anti-infection capabilities. Meanwhile, M2 macrophages utilize arginine to promote proline synthesis and collagen production, contributing to tissue repair and fibrosis 53-55. Macrophage amino acid metabolism regulates functional states through multiple pathways and is key to maintaining immune homeostasis and tissue health. Understanding and regulating macrophage amino acid metabolism, particularly glutamine metabolism, may offer new therapeutic approaches for CP.

#### 3.1.3 Lipid Metabolism

Lipid metabolism plays multiple roles in macrophage function, particularly in the polarization of M1 and M2 macrophages. Lipids serve not only as energy sources but also participate in cell signaling and gene regulation 56. In M1 macrophages, lipid metabolism is typically associated with pro-inflammatory responses. Excess lipid accumulation, particularly when fatty acid β-oxidation is enhanced, promotes the production of inflammatory cytokines such as TNF-α and IL-6, potentially amplifying the inflammatory response in CP 56.

Studies show that IL-4-activated M2 macrophages exhibit significantly upregulated fatty acid uptake and oxidation, while these functions are suppressed in M1 macrophages 57. M2 macrophages rely on lipid synthesis and storage to support their anti-inflammatory and tissue repair functions 57. In these cells, OXPHOS depends on the oxidation of fatty acids and glutamine, activating peroxisome proliferator-activated receptor-gamma (PPARγ), which regulates gene expression in M2 macrophages 58, 59. The natural ligand of PPARγ, 15-deoxy-Δ12,14-prostaglandin J2, inhibits NO, TNF-α, IL-1, and IL-6 production in monocytes and macrophages 60. PPARγ expression in macrophages is crucial for counteracting inflammation. It also inhibits NF-κB activity by physically interacting with NF-κB or inducing the expression of NF-κB inhibitors (IκB). For example, in the spontaneous CP model of WBN/Kob mice, PPARγ is highly expressed in macrophages. After treatment with PPARγ ligands, such as thiazolidinedione derivatives (TZD), the severity of pancreatic morphological damage is reduced, fibrosis is significantly improved, pancreatic amylase content decreases, serum IL-8 and TNF-α levels are reduced, and NF-κB binding activity decreases, preventing the exacerbation of CP 61, 62. In CP, M2 macrophages may contribute to tissue repair and fibrosis through lipid metabolism regulation. Therefore, targeting lipid metabolism pathways could become an effective strategy for controlling inflammation and promoting tissue repair in CP.

### 3.2 T Cell Metabolic Reprogramming

T cells play a vital role in defending against infections and in the immune response against tumors 63. T cells consist of various subtypes, each with distinct functions. During T cell activation, metabolic pathways undergo changes to provide sufficient energy and nutrients to support T cells' physiological functions. Once the immune response is completed, surviving T cells persist as memory T cells and revert to a quiescent oxidative metabolism state 64.

Regardless of the cause, CP is characterized by inflammatory cell infiltration, including lymphocytes and macrophages 65. Therefore, multiple studies suggest that the adaptive immune system plays a crucial role in CP pathogenesis, both in humans and animal models. Local chemokines recruit CD4^+^ and CD8^+^ T cells to CP lesions 66. Single-cell RNA sequencing of pancreatic immune cells from CP patients revealed that genetic CP is dominated by CD4^+^ helper T cell subpopulations, including CCR6^+^Th1, regulatory T cells (Tregs), and HLA-DA^+^CD8^+^ T cells. In contrast, idiopathic CP samples primarily exhibit FTH1^+^ CD4^+^ and BAG3^+^ CD8^+^ T cells 29. Peripheral blood samples from CP patients show an increased proportion of T-helper 1 cells (Th1), Th2, and Th17 compared to controls. Among alcohol-consuming patients, the proportion of Th1 cells is significantly higher than in non-drinkers, with CD4^+^ helper T cells infiltrating the pancreas mainly as Th1 and Th17 cells, and fewer Th2 cells 67. T cell response analysis in peripheral blood from CP patients, pancreatic cancer patients, and healthy individuals found that IL-10-specific responses in pancreatitis were mediated by IL-10^+^ IFN-γ- FoxP3^+^ regulatory T cells. Compared to pancreatic cancer, IL-10 levels in pancreatitis lesions are elevated, while interferon-γ (IFN-γ) levels are reduced, indicating that Tregs exhibit specific activity in pancreatitis 68.

T cells play a pivotal role in the inflammatory response and the progression of tissue damage in CP. Beyond the distinct distribution and functions of T cell subsets, metabolic changes within the immune microenvironment are also crucial in CP pathogenesis. Upon activation, T cells undergo metabolic reprogramming to meet their heightened energy demands and to support their functional roles. For instance, effector T cells (Teff) rely on glycolysis to sustain rapid proliferation and the production of inflammatory cytokines, while Tregs depend on fatty acid oxidation (FAO) to maintain their immunosuppressive function. The local metabolic environment within the pancreas of CP patients may regulate these metabolic pathways, thereby influencing T cell differentiation and function, and either exacerbating or alleviating the inflammatory response. Additionally, the activity of IL-10^+^ regulatory T cells in the pancreas could be closely linked to local metabolic signaling, offering new insights into the complex pathophysiology of chronic pancreatitis. Therefore, investigating the interaction between T cell metabolism and the pancreatic microenvironment is essential for developing novel therapeutic strategies.

#### 3.2.1 Glucose Metabolism

Glucose metabolism is critical for T cell activation and function. Studies show that during T cell activation, glucose demand increases significantly, primarily through upregulation of glucose transporters such as glucose transporter 1 (GLUT1) 69. T cells with specific GLUT1 deletion exhibit impaired CD4^+^T cell activation, clonal expansion, and survival 70. When glucose is scarce, CD8^+^ T cell function is inhibited, leading to reduced production of IFN-γ, granzyme, and perforin 71, 72.

Glycolysis not only provides energy for T cells but also regulates downstream effector functions, such as cytokine production, influencing T cell immune responses 73. Enhanced glycolysis can promote effector T cells (Teff) to produce more inflammatory cytokines, such as IFN-γ and TNF-α, which are drivers of chronic inflammatory responses 74. Glycolysis varies among different T cell subtypes: CD4^+^ T cells differentiate into Th1 and Th17 cells in a glycolysis-dependent manner, while Th2 cells exhibit higher glycolytic activity 75, 76. In CP and pancreatic fibrosis, T cell glycolysis may exacerbate inflammation, leading to tissue damage and fibrosis progression. This metabolic reprogramming not only affects immune cell activity but also regulates PSC activation through glucose metabolism-related signaling pathways, aggravating pancreatic fibrosis 6. Additionally, glucose metabolism changes may impact Tregs in the pancreas 77. Tregs play a vital role in maintaining tissue homeostasis and reducing inflammation, but glucose metabolism disorders may inhibit Treg activity, worsening the condition in CP.

Furthermore, T cell glucose metabolism reprogramming may influence their persistence and effector function in the pancreas. T cells with high glycolytic activity in chronic inflammation may exacerbate pancreatic fibrosis, as lactate promotes fibroblast activation and excessive extracellular matrix deposition 78, 79.

#### 3.2.2 Amino Acid Metabolism

Amino acid metabolism is essential for T cell proliferation and differentiation, particularly glutamine metabolism. Glutamine plays a crucial role in T cell activation by increasing the expression of transport proteins such as alanine-serine-cysteine transporter 2 (ASCT2) to facilitate glutamine uptake 80. Glutamine metabolism provides energy for T cells and regulates T cell function through the mTORC1 signaling pathway 81. Changes in amino acid metabolism are equally important in pancreatic diseases. Chronic inflammation leads to imbalances in amino acid metabolism, affecting T cells and other immune cells. For example, glutamine depletion can impair T cell polarization, exacerbating inflammation and fibrosis progression. Intermediate metabolites of glutamine metabolism, such as α-ketoglutarate, play roles in energy metabolism and may also influence gene expression through epigenetic modifications, such as DNA methylation, impacting T cell-mediated immune responses 82. This metabolic-epigenetic interaction may be crucial for maintaining chronic inflammation and exacerbating pancreatic fibrosis 50.

#### 3.2.3 Lipid Metabolism

T cell lipid metabolism exhibits significant variation in different functional states. Resting T cells primarily rely on fatty acid oxidation (FAO) to maintain energy balance, whereas effector T cells switch to glycolysis and lipid synthesis upon activation to support rapid proliferation and effector functions 83. Lipid metabolism is essential for T cell function, as activated T cells require large amounts of fatty acid synthesis to support cell membrane construction and signal transduction 75. In CP, lipid metabolism may influence T cell survival and function in the inflammatory microenvironment, contributing to disease progression. For example, increased FAO may help maintain the suppressive function of Tregs, partially limiting pancreatic inflammation 84. Further research suggests that changes in lipid metabolism pathways may exacerbate inflammation and fibrosis by affecting lipid deposition in pancreatic tissue 85.

The reprogramming of these metabolic pathways forms the foundation for T cell functional changes and plays a critical role in the inflammatory response and fibrosis progression in CP. This metabolic-immune interaction presents potential targets for developing new therapeutic strategies against chronic pancreatitis.

## 4. Metabolic Reprogramming in Pancreatic Stellate Cells

**Figure 2:**
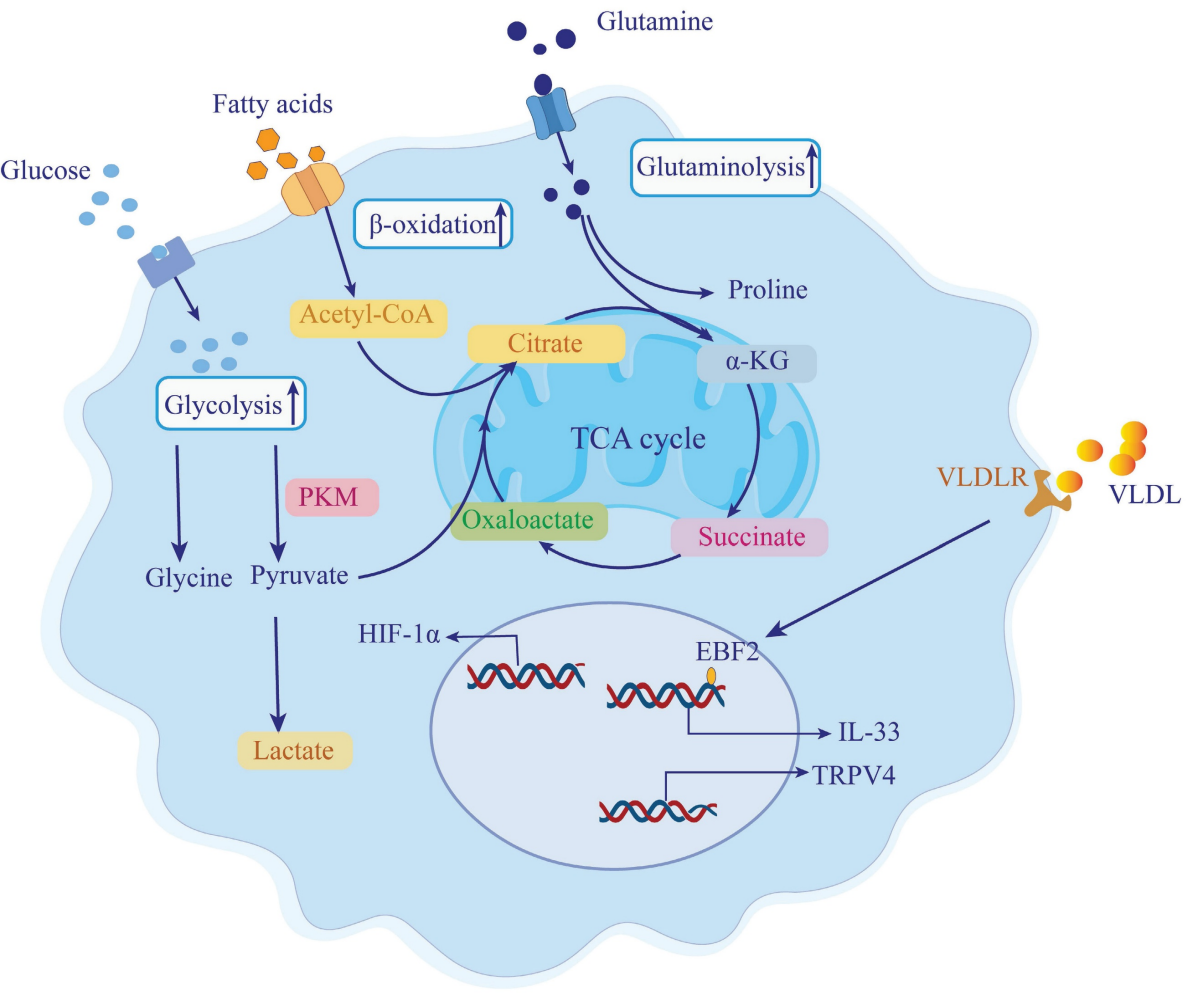
** Metabolic reprogramming of pancreatic stellate cells in chronic pancreatitis-associated fibrosis.** During chronic pancreatitis (CP), pancreatic stellate cells (PSCs) are activated by inflammatory and fibrogenic stimuli, undergoing a profound metabolic shift to support their transformation into myofibroblast-like cells. These activated PSCs exhibit enhanced glycolysis, lipid β-oxidation, and glutaminolysis to meet elevated bioenergetic and biosynthetic demands. Hypoxia, a common feature of fibrotic pancreatic tissue, induces the stabilization of hypoxia-inducible factor 1-alpha (HIF-1α), which promotes glycolytic flux and facilitates glycine biosynthesis for collagen production. Pyruvate kinase M2 (PKM2) activity further supports this glycolytic shift, resulting in increased lactate accumulation. Glutamine uptake and metabolism via glutaminase-1 (GLS1) generate glutamate and α-ketoglutarate (α-KG), fueling the tricarboxylic acid (TCA) cycle and driving proline synthesis, a critical amino acid for collagen assembly. Concurrently, fatty acid uptake and β-oxidation contribute acetyl-CoA to the TCA cycle, further amplifying metabolic flux. Additionally, lipid signaling plays a role in PSC activation. Very-low-density lipoproteins (VLDL) bind to their receptors (VLDLR) on PSC membranes, upregulating early B-cell factor 2 (EBF2) and stimulating the release of IL-33, a cytokine that activates type 2 immune responses. TRPV4 expression is also enhanced, linking lipid metabolism to mechanotransduction and promoting fibrogenic gene expression. These coordinated metabolic programs endow PSCs with sustained proliferative and fibrotic capacity, highlighting potential therapeutic targets to disrupt fibrosis progression in CP. PKM, pyruvate kinase M; HIF-1α, hypoxia-inducible factor-1 alpha; α-KG, α-ketoglutarate; TCA, tricarboxylic acid cycle; VLDL, very low-density lipoprotein; VLDLR, very low-density lipoprotein receptor; TRPV4, transient receptor potential vanilloid type 4; EBF2, early B-cell factor 2; GLS1, glutaminase 1.

Pancreatic fibrosis is a key pathological feature of CP, playing a critical role in the disease's progression 86, 87. PSCs are mesenchymal cells that account for only 4-7% of all parenchymal cells in a healthy pancreas and exist in a quiescent state. These cells contain lipid droplets rich in vitamin A 88, 89. The activation of PSCs is a crucial factor in pancreatic fibrosis. Once activated, PSCs transform into a myofibroblast-like phenotype, characterized by increased expression of α-smooth muscle actin (α-SMA), loss of lipid droplets, enhanced secretion of cytokines and ECM components, and increased migration and proliferation capabilities 90, 91. The sustained activation of PSCs leads to significant ECM overproduction, resulting in an imbalance between ECM synthesis and degradation, ultimately leading to fibrosis 92, 87, 31. An in-depth exploration of the metabolic reprogramming of PSCs could provide valuable insights into the underlying mechanisms of fibrosis in CP [Figure 2].

### 4.1 Increased Glycolysis

PSCs are activated in response to pancreatic tissue injury and stress, acquiring a myofibroblast-like state. This activation alters PSC metabolism, elevating glycolysis levels in CP and enhancing the Warburg effect through HIF-1α, thus promoting CP progression 93. During fibroblast differentiation into myofibroblasts, aerobic glycolysis is the primary energy source 94. In fibrosis, aerobic glycolysis provides the necessary ATP, amino acids, and nucleotides for ECM synthesis 95. Inhibiting aerobic glycolysis can suppress fibroblast activation and fibrosis 96. For example, myofibroblasts produce excess glycine during fibrosis to meet the demand for collagen production and secretion, and this glycine resynthesis depends on glycolysis. Glycolytic intermediates such as 3-phosphoglycerate are converted into serine, which is then converted into glycine 97, 98. PKM converts phosphoenolpyruvate (PEP) into pyruvate during glycolysis, and PKM2 reduces pyruvate kinase activity, promoting the accumulation of glycolytic intermediates, inducing glycine synthesis, and enhancing collagen secretion and expression 99. Using the PKM2 activator TEPP46 to convert PKM2 dimers into tetramers reduces serine and glycine expression in fibrosis models, decreasing collagen deposition in the pancreas and reversing cerulein-induced fibrosis 99. Resveratrol (RSV) inhibits PSC activation by suppressing miR-21, reducing lactate, Glut1, hexokinase 2 (HK2), PKM2, and lactate dehydrogenase A (LDHA) expression, thus blocking ROS-induced glycolysis in PSCs 100.

### 4.2 Amino Acid Metabolism

The proliferation of PSCs relies on glutamine catabolism, which provides the necessary bioenergetic and biosynthetic resources for activated PSCs. These activated PSCs exhibit elevated levels of glutaminase 1 (GLS1), a key enzyme in glutamine metabolism. High GLS1 expression enhances glutamine catabolism, resulting in increased production of α-KG 101. α-KG enters the tricarboxylic acid (TCA) cycle, boosting ATP production and supporting the synthesis of lipids, amino acids, and nucleic acids 99. Additionally, α-KG can be enzymatically converted into succinate, and the elevated levels of succinate promote glycolysis and stabilize HIF-1α, which facilitates myofibroblast differentiation 102. Glutamine also contributes to collagen synthesis by providing proline, thereby promoting fibrogenesis 103. It is essential for maintaining the proliferative phenotype of PSCs, with Yap-Myc signaling regulating PSC transdifferentiation by modulating glutamine metabolism. Inhibition of mitochondrial respiration and PSC growth was observed with the Yap inhibitor verteporfin, which reduced the expression of GLS1, Col1α1, and MMP2 in PSCs. Similarly, the Myc inhibitor MYCi975 also impaired PSC growth and mitochondrial respiration 101.

### 4.3 Lipid Metabolism

Lipid metabolism plays an important role in PSC activation and pancreatic fibrosis. Triglycerides (TGs) are lipid components that are transported to tissue cells via very-low-density lipoproteins (VLDLs), where they undergo catabolism to produce glycerol and fatty acids. These fatty acids are further oxidized to produce ATP, carbon dioxide, and water 104. Compared to glycolysis, lipid metabolism generates a large amount of ATP. Studies have shown that lipid metabolism disorders occur in pancreatitis, with lipid accumulation exacerbating inflammation 105, 106. However, the precise mechanism between lipid metabolism and pancreatic fibrosis remains unclear.

Consuming a high-fat diet can elevate pancreatic free fatty acids (FFAs) and trigger lipid peroxidation processes 107. In mice fed a long-term high-fat diet, metabolites promote TRPV4 expression and enhance PSC activation 108. In mice specifically expressing the R122H mutant, a high-fat diet induces more severe pancreatitis, including greater DNA damage, apoptosis, and collagen deposition 109. Wistar rats fed special feed (MB-3, a high-protein, high-fat diet) for 12 weeks develop fibrosis 110. Recent studies have shown that increased VLDLR expression in PSCs and the uptake of TG-rich lipoproteins upregulate lipid metabolism, increase IL-33 secretion, and activate type 2 immune responses, thereby inducing PSC activation and ultimately causing pancreatic fibrosis 111.

In conclusion, abnormal glucose, amino acid, and lipid metabolism provide energy and nutrients to PSCs, promoting pancreatic fibrosis. Therefore, further research into energy metabolism irregularities in CP and identifying metabolic pathway targets could enhance treatment options.

## 5. Metabolic Reprogramming as a Potential Target for Fibrosis Therapy

**Figure 3:**
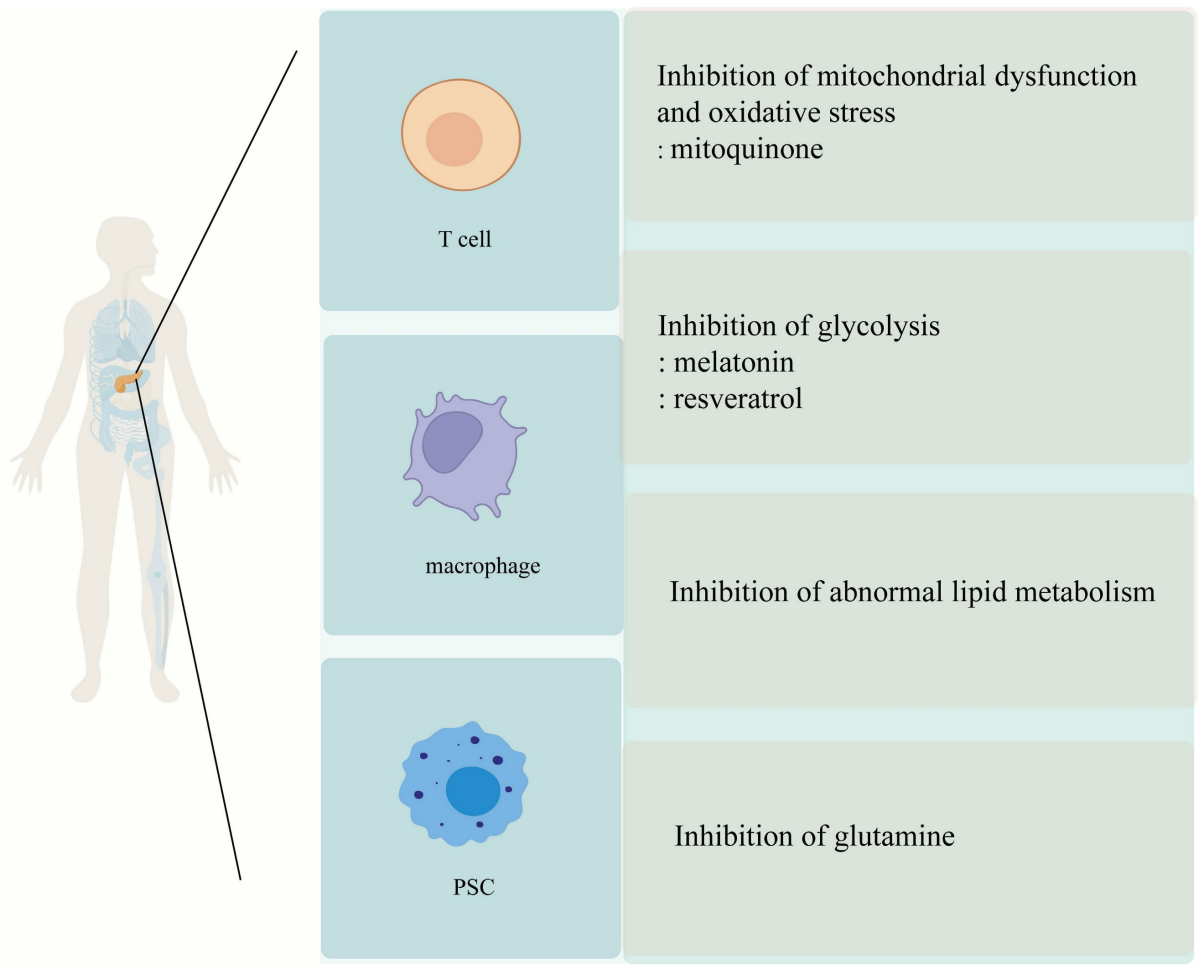
** Metabolism-targeted anti-fibrotic therapies in chronic pancreatitis.** This schematic highlights four therapeutic strategies targeting metabolic pathways in chronic pancreatitis (CP), applied across three key cell types involved in fibrosis: T cells, macrophages, and pancreatic stellate cells (PSCs). Mitochondrial-targeted antioxidants such as mitoquinone reduce oxidative stress and mitochondrial dysfunction, limiting ROS production, acinar cell death, and PSC activation. Glycolysis inhibitors like melatonin and resveratrol modulate macrophage and T cell phenotypes, suppressing inflammation (M1 to M2 polarization in macrophages) and reducing fibrosis-related PSC activation. Glutamine metabolism inhibitors target PSC metabolic reprogramming, limiting their activation, proliferation, and migration by disrupting key biosynthetic pathways such as α-ketoglutarate and proline synthesis. Finally, lipid metabolism modulation reduces fatty acid oxidation in PSCs and macrophages, limiting M2 macrophage polarization and decreasing PSC activation and ECM production. These therapies target distinct metabolic pathways but collectively address the metabolic vulnerabilities contributing to fibrogenesis in CP, with cell-specific targeting strategies offering the potential to minimize off-target effects and improve therapeutic efficacy. Abbreviations: PSC, pancreatic stellate cell; ROS, reactive oxygen species; M1, classically activated macrophage; M2, alternatively activated macrophage; α-KG, α-ketoglutarate; ECM, extracellular matrix.

Currently, there are no effective drugs or therapies for fibrosis associated with chronic pancreatitis. However, research has revealed that aberrant reprogramming of metabolic pathways not only contributes to persistent damage to pancreatic cells but also exacerbates the fibrosis process. Consequently, targeting metabolic reprogramming presents a promising new strategy for treating fibrosis in chronic pancreatitis. By modulating specific metabolic pathways, it may be possible to slow down or even reverse fibrosis progression, ultimately improving clinical outcomes for patients [Figure 3].

### 5.1 Regulation of Glucose Metabolism

Glycolysis is upregulated in both pancreatic stellate cells (PSCs) and pro-inflammatory immune cells under fibrotic conditions. Targeting glucose metabolism can disrupt the energy supply and biosynthetic pathways necessary for cell activation and ECM production, making it a common and viable antifibrotic strategy.

Glucose metabolism is central to the energy demands of activated PSCs in fibrotic tissues. Under hypoxic conditions, PSCs exhibit enhanced glycolysis, characterized by elevated expression of Glut1, phosphofructokinase (PFK), and lactate dehydrogenase (LDH). Melatonin, an indoleamine synthesized by the mammalian pineal gland at night, has protective effects in improving inflammation, oxidative stress, and pancreatic fibrosis 112. In PSCs treated with melatonin, glycolysis and glucose metabolism decrease. Melatonin regulates the MAPK and PI3K/AKT/mTOR signaling pathways, reducing PSC proliferation and viability. It also regulates the expression of mitochondrial fusion proteins responsible for cellular energy and metabolic performance 113. During pancreatic fibrosis, low oxygen utilization creates a hypoxic environment. Melatonin activates the p44/42/mTOR/p70S6K signaling pathway, inhibiting PSC proliferation and reducing α-SMA and type I collagen expression. Under hypoxic conditions, PSC glycolysis and glucose metabolism significantly increase, with elevated expression of Glut1, phosphofructokinase (PFK), and lactate dehydrogenase (LDH). After melatonin treatment, the expression of Glut-1, PFK, and LDH decreases. Therefore, melatonin inhibits PSC adaptation to mitochondrial energy supply under hypoxic conditions 114. Melatonin induces ROS production in mitochondria and the cytoplasm in a concentration-dependent manner, reducing PSC viability in a time- and concentration-dependent manner, leading to oxidative state changes in PSCs 115. Simultaneously, pharmacological concentrations of melatonin regulate the cell cycle, promote apoptosis, and reduce PSC proliferation under hypoxic conditions by activating caspase-3 and altering the expression of cyclins A and D 116. The melatonin receptor antagonist Luzindole regulates the p44/42/p38MAPKs signaling pathway, inducing endoplasmic reticulum stress and reducing the viability of rat pancreatic stellate cells 117. RSV, a natural polyphenol with multiple pharmacological effects, effectively inhibits H2O2-induced PSC activation, invasion, and migration by regulating miR-21 and suppressing ROS-induced PSC glycolysis. RSV also inhibits pancreatic cancer cell invasion and migration by suppressing ROS/miR-21 in PSCs 100. Hypoxia activates PSCs through HIF-1, and activated PSCs release IL-6, vascular endothelial growth factor A, and stromal cell-derived factor 1, promoting pancreatic cancer invasion, epithelial-mesenchymal transition, and inhibiting apoptosis. In the KPC mouse model, RSV inhibits hypoxia-induced PSC activation, blocking the interaction between PSCs and pancreatic cancer cells and disrupting the malignant evolution of pancreatic cancer 118.

Targeting glucose metabolism effectively limits PSC activation and reduces profibrotic signaling. Melatonin and RSV represent potent modulators that act on both energy production and inflammatory feedback, validating glycolysis as a therapeutic axis in fibrotic progression.

### 5.2 Targeting Amino Acid Metabolism

Amino acid metabolism supports redox balance, biosynthesis, and signaling in both PSCs and immune cells. Dysregulation in glutamine and cysteine pathways contributes to oxidative stress and fibrogenic transformation. Intervening in these pathways provides opportunities to disrupt multiple profibrotic mechanisms.

Amino acid imbalance is common in CP due to impaired exocrine function. Among amino acids, glutamine and cysteine metabolism are particularly relevant to PSC activation and redox homeostasis. In patients with CP, serum levels of essential and aromatic amino acids are reduced, possibly due to exocrine pancreatic dysfunction. The mechanisms linking amino acid metabolism to CP development are still under investigation. N-acetylcysteine (NAC) serves as a precursor for the biosynthesis of glutathione (GSH), an antioxidant with properties that can reduce oxidative stress. NAC supplementation increases glutathione levels and reduces oxidative stress. Additionally, NAC can inhibit PSCs activation, and when combined with pioglitazone, it helps maintain PSCs in a quiescent-like state 119. Glutathione, composed of glutamate, glycine, and cysteine, is an antioxidant that inhibits PSC activation and proliferation by blocking the ROS/TGFβ/SMAD signaling pathway, thereby reducing pancreatic fibrosis 120. Up-regulation of glutamine catabolism is important for PSC activation, proliferation, and migration, so inhibiting glutamine catabolism may be an effective way to inhibit pancreatic fibrosis. Yap-Myc signaling regulates glutamine catabolism to activate PSCs, and inhibition of glutamine catabolism inhibits cell growth and fibrosis formation 101. Glutamine in PSC promotes the biological function of cancer cells, and treatment with palmatine (PMT) for 48 h reduced the expression of GLI1 and GLI2 in PSC and downstream target COL1A1 expression, disrupting glutamine-mediated PCC action. PMT in combination with gemcitabine inhibited stellate cell and cancer cell growth 121.

Amino acid metabolism is essential to maintaining the redox state and biosynthetic demand of activated PSCs. Agents such as NAC and PMT interfere with glutamine and GSH pathways, providing both antifibrotic and anti-inflammatory benefits.

### 5.3 Targeting Lipid Metabolism

Lipid metabolism affects PSC activation through nuclear receptors like PPARγ and modulates immune cell polarization via fatty acid oxidation. Regulating lipid metabolism bridges stromal and immune axes in pancreatic fibrosis.

Lipid metabolic reprogramming influences both PSC activation and macrophage polarization in CP. PPARγ is a transcription factor that plays a critical role in adipogenesis. Exosomes derived from acinar cells, containing miR-13a-3p, act on PSCs by targeting PPARγ, leading to PSC activation and collagen synthesis, thus driving pancreatic fibrosis 122. Pioglitazone, a PPARγ agonist, reduces cerulein-induced pancreatitis, inhibits IL-1β production and release, and enhances pancreatic HSP70 expression 123. During the early stages of chronic pancreatitis, saturated fatty acids may inhibit fibrosis through the PERK pathway 124. Fatty acid metabolism provides energy for M2 macrophages. Short-chain fatty acids (SCFAs) produced by G+ bacteria help maintain intestinal barrier integrity, regulate M2 macrophage infiltration and polarization, and alleviate pancreatic fibrosis 125. Under oxidative stress conditions, fatty acid peroxidation increases MDA levels. Intervening in lipid metabolic disorders offers another therapeutic route to protect pancreatic tissue from fibrotic remodeling.

Lipid metabolic interventions—via PPARγ agonists, SCFAs, or saturated fatty acids—have pleiotropic effects on PSCs and immune cells. Their ability to suppress inflammation and ECM production highlights lipid regulation as a versatile antifibrotic approach.

### 5.4 Antioxidant Therapies

Oxidative stress is a shared upstream driver of PSC and immune cell activation. Targeting mitochondrial ROS and redox imbalance is a foundational strategy to disrupt fibrotic signaling.

Mitochondria are the primary source of ROS within cells 126. Using mitochondria-targeted antioxidants can significantly reduce oxidative stress (OS) 127, preventing OS from activating PSCs through the AMPK signaling pathway. This leads to the overexpression of α-SMA, enhanced cell proliferation, and migration 128, 129.

MitoQ is a well-studied mitochondria-targeted antioxidant and one of the most extensively researched compounds of its kind. Clinical studies on liver disease models have consistently shown that MitoQ is highly effective in treating various liver diseases 130-132. In a cerulein-induced acute pancreatitis mouse model, two intraperitoneal injections of MitoQ inhibited ROS production, preventing protective apoptosis and promoting pancreatic acinar cell death, worsening vacuole damage 133. Recently, another study demonstrated that oral administration of MitoQ downregulated fibrosis-related genes in CP mice, including type I collagen (Col I), type III collagen (Col III), and tissue inhibitor of metalloproteinases 1 (TIMP1). Additionally, SOD enzyme mRNA expression levels and activity were elevated in CP mice and HPSCs 134. Superoxide dismutase (SOD) catalyzes the reduction of superoxide ions to H_2_O_2_, which can be decomposed into water by peroxidases. Decreased SOD activity exacerbates ROS-induced damage, leading to increased levels of DNA, lipid, and protein damage caused by free radicals 126, 135. Therefore, when present in appropriate concentrations, MitoQ is expected to alleviate pancreatic fibrosis by reducing ROS levels and inhibiting PSC activation. Moreover, it reduces intracellular OS by enhancing SOD expression and activity, accelerating the clearance of oxygen free radicals. By reducing ROS levels, enhancing antioxidant defenses, and inhibiting PSC activation, MitoQ and related compounds hold potential as adjunct therapies for fibrosis prevention.

Mitochondria-targeted antioxidants like MitoQ, by restoring redox homeostasis, inhibit fibrotic pathways common to PSCs and immune cells. Antioxidant therapy serves as a critical upstream intervention in fibrosis prevention.

## 6. Conclusion

CP is marked by a complex interplay of cellular and molecular mechanisms that lead to pancreatic fibrosis, significantly impacting patients' quality of life and increasing their risk of developing pancreatic cancer 136. The recent focus on metabolic reprogramming in PSCs and immune cells offers promising avenues for therapeutic intervention. By understanding how glucose, lipid, and amino acid metabolism contribute to fibrosis, we can identify potential targets to slow or reverse disease progression.

The reprogramming of glucose metabolism, particularly the enhancement of glycolysis in PSCs, is a critical factor in fibrosis. Targeting key glycolytic enzymes such as PKM2 could reduce excessive extracellular matrix production and fibrosis. Similarly, modulating lipid metabolism, which provides energy for PSCs and immune cells during chronic inflammation, presents another potential strategy. The role of fatty acid oxidation and the activation of PPARγ in reducing fibrosis highlights the therapeutic potential of targeting lipid metabolism.

Amino acid metabolism, especially glutamine metabolism, also plays a crucial role in PSC and immune cell function, influencing fibrosis through the provision of essential metabolites and the regulation of signaling pathways. Inhibiting glutamine metabolism may help reduce fibrosis and improve clinical outcomes.

As we advance in the exploration of metabolic reprogramming in CP, we open up promising avenues for the development of innovative therapeutic strategies. Future research should focus on elucidating the molecular mechanisms that drive metabolic alterations in PSCs and immune cells, with an emphasis on translating these insights into effective clinical interventions. Combining targeted metabolic inhibitors with existing anti-inflammatory and antioxidant therapies could offer a more comprehensive approach to managing CP, potentially decelerating disease progression and enhancing patient outcomes.

Furthermore, as we deepen our understanding of the intricate metabolic changes in CP, there is a significant opportunity to discover biomarkers that could predict disease progression and therapeutic response. These biomarkers could be instrumental in advancing personalized medicine, allowing for more tailored and effective treatment strategies for patients suffering from chronic pancreatitis.

In summary, integrating metabolic insights into the treatment of CP holds immense potential to revolutionize the therapeutic landscape of this challenging disease, providing new hope for better management and an improved quality of life for patients.
